# Prognostic Significance of Sentinel Lymph Node Status in Thick Primary Melanomas (> 4 mm)

**DOI:** 10.1245/s10434-023-14050-w

**Published:** 2023-08-14

**Authors:** Carl-Jacob Holmberg, Rasmus Mikiver, Karolin Isaksson, Christian Ingvar, Marc Moncrieff, Kari Nielsen, Lars Ny, Johan Lyth, Roger Olofsson Bagge

**Affiliations:** 1 Department of Surgery, Sahlgrenska University Hospital, Gothenburg, Sweden; 2 Department of Surgery, Sahlgrenska Center for Cancer Research, Institute of Clinical Sciences, Sahlgrenska Academy, University of Gothenburg, Gothenburg, Sweden; 3 Wallenberg Centre for Molecular and Translational Medicine, University of Gothenburg, Gothenburg, Sweden; 4 Department of Clinical and Experimental Medicine, Regional Cancer Center Southeast Sweden, Linköping University, Linköping, Sweden; 5 Department of Surgery, Kristianstad Hospital, Kristianstad, Sweden; 6 Division of Surgery, Department of Clinical Sciences, Lund University, Lund, Sweden; 7 Lund University Cancer Centre, Lund University, Lund, Sweden; 8 Department of Plastic and Reconstructive Surgery, Norfolk and Norwich University Hospital, Norwich, UK; 9 Department of Dermatology, Skåne University Hospital, Lund, Sweden; 10 Division of Dermatology, Department of Clinical Sciences, Lund University, Lund, Sweden; 11 Department of Oncology, Institute of Clinical Sciences, Sahlgrenska Academy, University of Gothenburg, Sahlgrenska University Hospital, Gothenburg, Sweden; 12 Department of Health, Medicine and Caring Sciences, Linköping University, Linköping, Sweden

## Abstract

**Background:**

The key prognostic factors for staging patients with primary cutaneous melanoma are Breslow thickness, ulceration, and sentinel lymph node (SLN) status. The multicenter selective lymphadenectomy trial (MSLT-I) verified SLN status as the most important prognostic factor for patients with intermediate-thickness melanoma (Breslow thickness, 1–4 mm). Although most international guidelines recommend SLN biopsy (SLNB) also for patients with thick (> 4 mm, pT4) melanomas, its prognostic role has been questioned. The primary aim of this study was to establish whether SLN status is prognostic in T4 melanoma tumors.

**Methods:**

Data for all patients with a diagnosis of primary invasive cutaneous melanoma of Breslow thickness greater than 1 mm in Sweden between 2007 and 2020 were retrieved from the Swedish Melanoma Registry, a large prospective population-based registry. A multivariable Cox proportional hazard model for melanoma-specific survival (MSS) was constructed based on Breslow thickness stratified for SLN status.

**Results:**

The study enrolled 10,491 patients, 1943 of whom had a Breslow thickness greater than 4 mm (pT4). A positive SLN was found for 34% of these pT4 patients. The 5-year MSS was 71%, and the 10-year MSS was 62%. There was a statistically significant difference in MSS between the patients with a positive SLN and those with a negative SLN (hazard ratio of 2.4 (95% confidence interval CI 1.6–3.5) for stage T4a and 2.0 (95% CI 1.6–2.5) for satage T4b.

**Conclusion:**

Sentinel lymph node status gives important prognostic information also for patients with thick (> 4 mm) melanomas, and the authors thus recommend that clinical guidelines be updated to reflect this.

**Supplementary Information:**

The online version contains supplementary material available at 10.1245/s10434-023-14050-w.

Melanoma has a rapidly increasing incidence, especially in fair-skinned populations common to the Nordic countries.^1^ In Sweden, incidence rates continuously rise by as much as 4–6% annually, and melanoma currently is the fifth most common malignancy.^2,3^ The most important prognostic factors of the disease are Breslow thickness, ulceration status, and sentinel lymph node (SLN) status.^4,5^ The sentinel lymph node biopsy (SLNB) technique introduced by Morton et al.^6^ is a minimally invasive and low-morbidity surgical procedure in which the first draining lymph node is identified, removed, and analyzed for the presence of metastasis. This allows for the detection of subclinical metastatic disease.

The literature shows several reported predictive factors for SLN positivity including Breslow thickness, ulceration status, mitotic rate, age, primary tumor site, lympho-vascular invasion (LVI), presence of tumor-infiltrating lymphocytes (TIL), and sex.^7^ The multicenter selective lymphadenectomy trial (MSLT-I) was the landmark phase 3 clinical trial that compared the outcome of SLNB with nodal observation. The study validated SLN status as the most important prognostic factor, together with Breslow thickness and ulceration, for patients with intermediate-thickness primary cutaneous melanoma (1–4 mm, American Joint Committee on Cancer [AJCC] stage T2–T3).^8^ As a result, SLN status is an integral part of the AJCC classification system for cutaneous melanoma.

For patients with thick primary melanomas (> 4 mm, AJCC stage T4), the prognostic significance of SLN status has been less clear. According to the current joint American Society of Clinical Oncology (ASCO) and Society of Surgical Oncology (SSO) clinical practice guideline, as well as National Comprehensive Cancer Network (NCCN) guidelines, SLNB “may be recommended” for patients with thick melanoma for the purposes of staging, referral for adjuvant systemic therapy, and potentially for regional disease control, which is a weaker recommendation than for melanomas with 1–4 mm of thickness (stage T2–T3 tumors), for which SLNB is “recommended.”^9,10^

The primary aim of the current study was to establish whether SLN status is prognostic also for T4 tumors. The secondary aims were to examine whether the prognostic value of SLN status changes with increasing Breslow thickness, and to verify known factors predictive for SLN status.

## Patients and Methods

### Patients

Data for all patients with a first diagnosis of primary invasive cutaneous melanoma (AJCC 8th-edition stage pT2a–pT4b) in Sweden between 1 January 2007 and 31 December 2020 were retrieved from the national Swedish Melanoma Registry (SweMR). The study excluded patients with a Breslow tumor thickness of 1 mm or less, patients for whom SLNB was not performed, patients with locoregional and distant metastases (stages III and IV) at the time of the initial diagnosis, and patients with missing information on SLN status.

For the patients with multiple subsequent melanomas, survival was calculated from diagnosis of the primary melanoma but censured starting from the time a second melanoma was diagnosed. Thus, such patients could contribute survival data, but only for their primary melanoma.

**Fig. 1:**
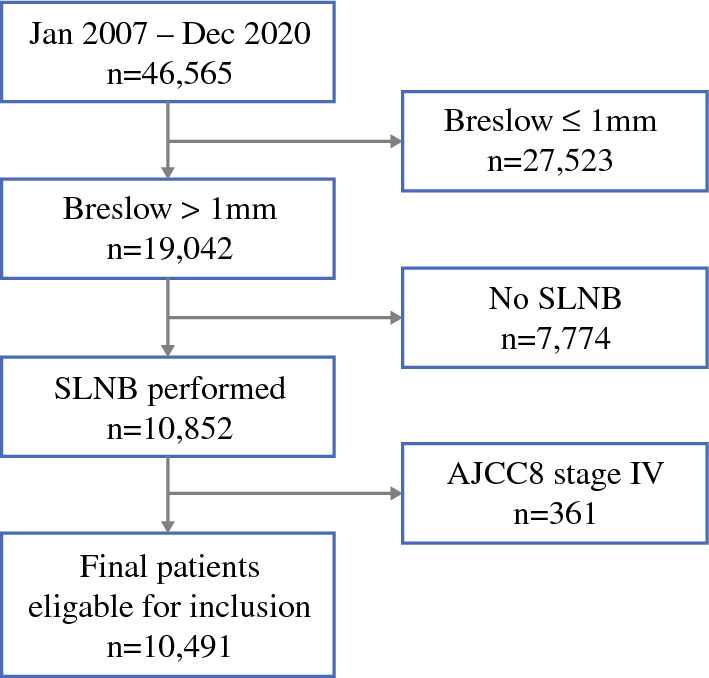
Flowchart of the included melanoma patients

For the final analysis, 10,491 patients with a first primary invasive cutaneous melanoma pT2a–pT4b were eligible and included (Fig. 1). Clinical and histopathologic variables including age, sex, Breslow thickness, ulceration, mitotic rate, histopathologic subtype, diagnosis period, tumor site, and SLN status were retrieved. Patients were staged according to the American Joint Committee (AJCC) eighth edition,^11^ and for patients whose disease stage data were originally registered according to earlier AJCC editions, the data were re-coded in accordance with the eighth edition. Information on the date and cause of death was obtained from the Swedish Cause of Death Registry, which was linked to SweMR to calculate melanoma-specific survival (MSS). Information concerning race/ethnicity is not available in the registry. All the patients were followed until death, until censored at diagnosis of a subsequent melanoma, or until the end of follow-up period on 31 December 2020.

The SweMR is a nationwide, population-based registry that includes prospectively collected clinicopathologic data on invasive cutaneous melanoma in Sweden since 1990. Coverage has been high (99%) since 1996. The registry was initiated by the multidisciplinary Swedish Melanoma Study Group.

### Statistical Evaluation

Statistical analysis was performed using a multivariable Cox proportional hazard model for MSS and reported as a hazard ratio (HR) for death based on Breslow thickness. Kaplan–Meier (KM) curves were used to illustrate MSS for negative and positive SLN status stratified by Breslow thickness and AJCC T stage. A logistic regression analysis was performed to identify significant independent predictive factors for SLN status. Multivariable analyses were based on complete patient data and excluded patients with missing data. Continuous variables were presented as median with interquartile range (IQR). Statistical significance was set at *p* value lower than 0.05. All analyses were performed using R version 3.5.1 (The R Foundation for Statistical Computing). The study was approved by the Swedish Ethical Review Authority (Dnr 99/160) and performed in accordance with the Declaration of Helsinki.

## Results

From the registry, 10,491 patients with melanoma of Breslow thickness greater than 1 mm who underwent SLNB were identified (Table 1). In this overall cohort, 54% (*n* = 5668) were males, the overall median age was 65 years (IQR, 53–73 years), and the overall median Breslow thickness was 2 mm (IQR, 1.3–3.5 mm). The overall most common histopathologic subtype was superficial spreading melanoma (SSM) (53%, *n* = 5504), followed by nodular melanoma (NM) (30%, *n* = 3116). Ulceration of the primary melanoma was present in 34% (*n* = 3552) of the patients. The distribution of the primary melanoma sites was as follows; trunk 43% (*n* = 4457), extremities 48% (*n* = 5046), head/neck 7% (*n* = 764), and palm/subungual 2% (*n* = 208).

**Table 1:** Patient and primary tumor characteristics

Tumor thickness (mm)	1.1–2.0	2.1–3.0	3.1–4.0	4.1–5.0	5.1–6.0	6.1–7.0	7.1–8.0	>8.0	All
	(*n* = 5407) *n* (%)	(*n* = 2072) *n* (%)	(*n* = 1069) *n* (%)	(*n* = 765) *n* (%)	(*n* = 374) *n* (%)	(*n* = 232) *n* (%)	(*n* = 145) *n* (%)	(*n* = 427) *n* (%)	(*n* = 10,491) *n* (%)
*Sex*									
Men	2710 (50.1)	1131 (54.6)	622 (58.2)	484 (63.3)	236 (63.1)	140 (60.3)	88 (60.7)	257 (60.2)	5668 (54.0)
Women	2697 (49.9)	941 (45.4)	447 (41.8)	281 (36.7)	138 (36.9)	92 (39.7)	57 (39.3)	170 (39.8)	4823 (46.0)
Age (IQR)	62.0 (50.0–71.0)	65.0 (54.0–73.0)	67.0 (56.0–74.0)	69.0 (60.0–76.0)	70.0(60.0–76.0)	72.0 (62.8–77.0)	72.0 (61.0–79.0)	68.0 (60.0–76.0)	65.0 (53.0–73.0)
*Age (years)*									
< 40	537 (9.9)	159 (7.7)	54 (5.1)	36 (4.7)	18 (4.8)	10 (4.3)	4 (2.7)	17 (4.0)	835 (8.0)
40–59	1889 (34.9)	580 (28.0)	294 (27.5)	154 (20.1)	75 (20.1)	34 (14.7)	27 (18.6)	88 (20.6)	3141 (29.9)
60–69	1440 (26.6)	554 (26.7)	278 (26.0)	193 (25.2)	86 (23.0)	50 (21.6)	33 (22.8)	129 (30.2)	2763 (26.3)
70–79	1243 (23.0)	586 (28.3)	328 (30.7)	271 (35.4)	137 (36.6)	101 (43.5)	48 (33.1)	138 (32.3)	2852 (27.2)
≥ 80	298 (5.5)	193 (9.3)	115 (10.8)	111 (14.5)	58 (15.5)	37 (15.9)	33 (22.8)	55 (12.9)	900 (8.6)
*Tumor site*									
Extremities	2756 (51.0)	981 (47.3)	486 (45.5)	317 (41.4)	163 (43.6)	106 (45.7)	65 (44.8)	172 (40.3)	5046 (48.1)
Head/neck	337 (6.2)	152 (7.3)	83 (7.7)	74 (9.7)	43 (11.5)	23 (9.9)	14 (9.7)	38 (8.9)	764 (7.3)
Trunk	2251 (41.6)	883 (42.6)	472 (44.2)	343 (44.8)	154 (41.2)	95 (40.9)	61 (42.1)	198 (46.4)	4457 (42.5)
Palm/subungual	58 (1.1)	52 (2.5)	26 (2.4)	29 (3.8)	13 (3.5)	7 (3.0)	5 (3.5)	18 (4.2)	208 (2.0)
Missing	5 (0.1)	4 (0.2)	2 (0.2)	2 (0.3)	1 (0.3)	1 (0.4)	0 (0.0)	1 (0.2)	16 (0.2)
*Tumor ulceration*									
Absent	4294 (79.4)	1201 (58.0)	483 (45.2)	295 (38.6)	134 (35.8)	71 (30.6)	42 (29.0)	111 (26.0)	6631 (63.2)
Present	944 (17.5)	806 (38.9)	555 (51.9)	459 (60.0)	233 (62.3)	154 (66.4)	98 (67.6)	303 (71.0)	3552 (33.9)
Missing	169 (3.1)	65 (3.1)	31 (2.9)	11 (1.4)	7 (1.9)	7 (3.0)	5 (3.5)	13 (3.0)	308 (2.9)
*Histopathologic subtype*									
SSM	3698 (68.4)	982 (47.4)	373 (34.9)	208 (27.2)	99 (26.5)	43 (18.5)	30 (20.7)	71 (16.6)	5504 (52.5)
ALM	58 (1.1)	34 (1.6)	25 (2.3)	22 (2.9)	10 (2.7)	4 (1.7)	3 (2.07)	20 (4.7)	176 (1.7)
NM	734 (13.6)	748 (36.1)	524 (49.0)	404 (52.8)	205 (54.8)	144 (62.1)	90 (62.1)	267 (62.5)	3116 (29.7)
Other	701 (13.0)	224 (10.8)	112 (10.5)	105 (13.7)	47 (12.6)	36 (15.5)	16 (11.0)	59 (13.8)	1300 (12.4)
Missing	95 (1.8)	43 (2.1)	23 (2.2)	18 (2.4)	8 (2.1)	5 (2.2)	3 (2.1)	5 (1.2)	200 (1.9)
*SLN status*									
Positive	561 (10.4)	470 (22.7)	305 (28.5)	243 (31.8)	112 (29.9)	84 (36.2)	61 (42.1)	168 (39.3)	2004 (19.1)
Negative	4846 (89.6)	1602 (77.3)	764 (71.5)	522 (68.2)	262 (70.1)	148 (63.8)	84 (57.9)	259 (60.7)	8487 (80.9)

IQR, interquartile range; SSM, superficial spreading melanoma; ALM, acral lentiginous melanoma; NM, nodular melanoma

The focus of the analysis was on 1943 patients (19%) who had a thick melanoma, with a Breslow thickness greater than 4 mm (pT4). Of these 1943 patients, 1205 (62%) were males. The median age was 70 years (IQR, 60–76 years), and the median Breslow thickness was 5.7 mm (IQR, 4.8–8.0 mm). The most common histopathologic subtype in this group was NM (57%, *n* = 1110), followed by SSM (23%, *n* = 451). Ulceration of the primary melanoma was present in 64% (*n* = 1247) of the patients. The distribution of the primary melanoma sites was as follows; trunk 44% (*n* = 851), extremities 42% (*n* = 823), head/neck 10% (*n* = 192), and palm/subungual 4% (*n* = 72).

### Melanoma-Specific Survival

**Fig. 2:**
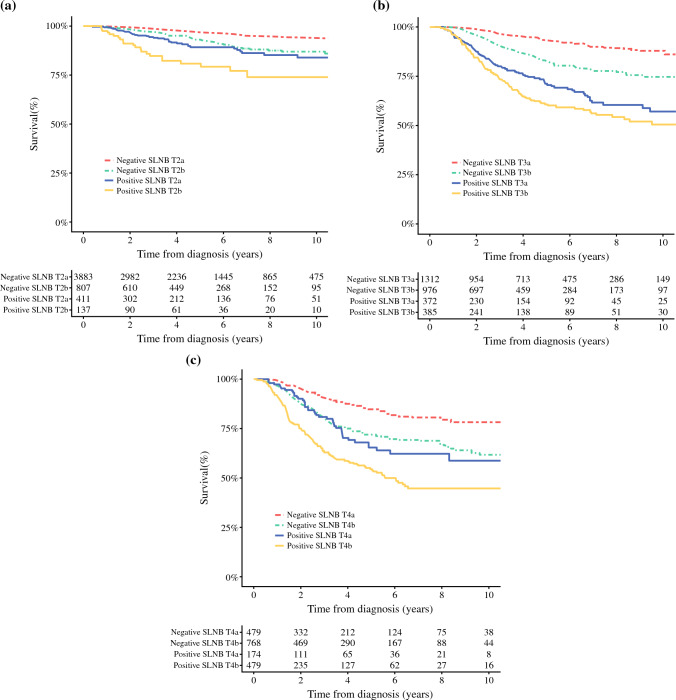
Melanoma-specific survival of patients with (**a**) stage T2, (**b**) stage T3, and (**c**) stage T4 tumors as stratified by sentinel lymph node (SLN) status

For the patients with T4 melanomas, the 5-year MSS was 71% (95% CI 68–73%), and the 10-year MSS was 62% (95% CI 59–66%), with the median MSS not reached. When the T4 patients were stratified by SLN status, the 5-year MSS rates for the SLN-positive and SLN-negative patients were respectively 57% (95% CI 53–63%) and 77% (95% CI 75–80%), whereas the 10-year MSS rates were respectively 49% (95% CI 43–55%) and 69% (95% CI 64–73%) (Fig. 2c). The independent prognostic factors for MSS were SLN status, age older than 80 years, tumor ulceration, and Breslow thickness.

The 5-year MSS for the entire cohort (Breslow thickness, > 1 mm) was 87% (95% CI 87–88%), and the 10-year MSS was 82% (95% CI 81–83%), although the median MSS was not reached. The independent prognostic factors for MSS were age, SLN status, Breslow thickness, and tumor ulceration (Table 2). For the SLN-negative patients overall, the 5- and 10-year MSS rates were respectively 92% (95% CI 91–92%) and 87% (95% CI 85–88%), whereas for the SLN-positive patients overall, the 5- and 10-year MSS rates were respectively 69% (95% CI 67–72%) and 60% (95% CI 57–64%) (HR 4.0 (95% CI, 3.6–4.5); *p* < 0.0001; Fig. S1a and b). When stratified by T stage, the survival of the patients with a positive SLN was consistently lower than the survival of those with a negative SLN (Fig. 2a–c).

**Table 2:** Multivariable analysis of prognostic factors for melanoma-specific survival (*n* = 9878)

Breslow thickness (mm)	1.1–2.0	2.1–3.0	3.1–4.0	4.1–5.0	5.1–6.0	6.1–7.0	7.1–8.0	>8	All
	(*n* = 5407) HR (95% CI))	(*n* = 2072) HR (95% CI)	(*n* = 1069) HR (95% CI)	(*n* = 765) HR (95% CI)	(*n* = 374) HR (95% CI)	(*n* = 232) HR (95% CI)	(*n* = 145) HR (95% CI)	(*n* = 427) HR (95% CI)	(*n* = 10,491) HR (95% CI)
*Outcome of SLNB*									
Negative	1 (ref)	1 (ref)	1 (ref)	1 (ref)	1 (ref)	1 (ref)	1 (ref)	1 (ref)	1 (ref)
Positive	3.1 (2.3–4.3)	3.6 (2.8–4.8)	2.8 (2.1–3.8)	1.8 (1.2–2.6)	2.5 (1.5–4.4)	1.4 (0.8–2.7)	3.5 (1.6–7.9)	2.8 (2.0–4.1)	2.8 (2.5–3.2)
*Sex*									
Men	1 (ref)	1 (ref)	1 (ref)	1 (ref)	1 (ref)	1 (ref)	1 (ref)	1 (ref)	1 (ref)
Women	0.6 (0.5–0.8)	0.7 (0.5–1.0)	0.6 (0.4–0.9)	0.7 (0.5–1.1)	0.4 (0.2–0.8)	0.4 (0.2–0.7)	0.5 (0.3–1.1)	1.4 (1.0–2.1)	0.7 (0.6–0.8)
*Age (years)*									
< 40	1 (ref)	1 (ref)	1 (ref)	1 (ref)	1 (ref)	1 (ref)	1 (ref)	1 (ref)	1 (ref)
40–59	1.1 (0.7–1.8)	1.4 (0.8–2.5)	1.3 (0.6–3.0)	0.8 (0.3–2.0)	2.4 (0.6–9.3)	0.4 (0.1–1.5)	0.3 (0–1.8)	1.4 (0.4–4.7)	1.1 (0.8–1.5)
60–69	1.2 (0.7–2.0)	1.8 (1.0–3.2)	1.8 (0.8–4.0)	1.2 (0.5–2.9)	3.6 (1.0–13.1)	1.4 (0.4–5.2)	0.1 (0–0.9)	1.9 (0.6–6.2)	1.5 (1.1–2.0)
70–79	1.5 (0.9–2.5)	1.7 (0.9–3.0)	2.2 (1.0–4.9)	1.3 (0.5–3.1)	2.7 (0.8–9.4)	0.6 (0.2–2.1)	0.2 (0–1.3)	2.1 (0.6–6.9)	1.6 (1.2–2.1)
≥ 80	2.7 (1.4–5.0)	2.7 (1.4–5.4)	2.7 (1.1–6.5)	1.4 (0.5–3.8)	5.8 (1.5–22.6)	0.4 (0.1–1.8)	0.3 (0–1.6)	3.2 (0.9–10.9)	2.2 (1.6–3.0)
*Tumor site*									
Extremities	1 (ref)	1 (ref)	1 (ref)	1 (ref)	1 (ref)	1 (ref)	1 (ref)	1 (ref)	1 (ref)
Head/neck	3.1 (1.9–4.9)	1.0 (0.5–2.0)	0.7 (0.3–1.5)	0.5 (0.2–1.3)	1.2 (0.4–3.2)	0.5 (0.2–1.6)	2.6 (0.8–8.7)	0.6 (0.2–1.4)	1.1 (0.9–1.5)
Trunk	1.3 (0.9–1.7)	1.1 (0.8–1.4)	1.3 (0.9–1.7)	0.9 (0.6–1.3)	1.0 (0.6–1.8)	0.7 (0.4–1.3)	0.7 (0.3–1.5)	1.3 (0.9–1.9)	1.1 (1.0–1.3)
Palm/subungual	1.2 (0.3–4.6)	1.4 (0.6–3.0)	1.7 (0.6–4.7)	0.9 (0.3–2.4)	1.8 (0.6–5.9)	1.1 (0.1–19.2)	–	0.6 (0.2–2.1)	1.1 (0.7–1.6)
*Tumor ulceration*									
Absent	1 (ref)	1 (ref)	1 (ref)	1 (ref)	1 (ref)	1 (ref)	1 (ref)	1 (ref)	1 (ref)
Present	2.1 (1.6–2.7)	1.7 (1.3–2.3)	1.4 (1.0–1.9)	1.6 (1.1–2.3)	1.9 (1.0–3.5)	2.1 (1.0–4.4)	0.9 (0.4–2.0)	2.4 (1.4–3.9)	1.8 (1.5–2.0)
*Histopathologic subtype*									
SSM	1 (ref)	1 (ref)	1 (ref)	1 (ref)	1 (ref)	1 (ref)	1 (ref)	1 (ref)	1 (ref)
ALM	0.7 (0.1–3.5)	1.2 (0.5–3.0)	1.2 (0.4–3.5)	0.6 (0.2–1.8)	2.0 (0.5–7.9)	0.6 (0–10.9)	–	1.2 (0.4–3.5)	1.0 (0.6–1.6)
NM	1.1 (0.8–1.6)	0.7 (0.6–1.0)	1.3 (0.9–1.8)	0.6 (0.4–0.9)	1.5 (0.8–2.8)	0.6 (0.3–1.3)	0.7 (0.3–1.6)	1.0 (0.6–1.6)	0.9 (0.8–1.1)
Other	0.6 (0.4–1.0)	0.4 (0.2–0.8)	0.8 (0.4–1.4)	0.8 (0.5–1.4)	1.8 (0.8–4.1)	0.5 (0.2–1.6)	0.7 (0.2–2.1)	0.8 (0.4–1.5)	0.7 (0.6–0.9)

HR, hazard ratio; CI, confidence interval; SLNB, sentinel lymph node biopsy; SSM, superficial spreading melanoma; ALM, acral lentiginous melanoma; NM, nodular melanoma

### SLN Status

For the overall cohort, the rate of positive SLN status was 19% (*n* = 2004). The predictive factors for a positive SLN status overall were increasing Breslow thickness, age, tumor site, tumor ulceration, and histopathologic subtype. For the T4 patients, the rate of positive SLN status was 34% (*n* = 668). The predictive factors for a positive SLN status in the multivariable analysis were increasing Breslow thickness, presence of ulceration, increasing Clark´s level of invasion, age, tumor site (increased odds ratio [OR] for trunk and palm/subungual localization and decreased OR for head and neck), and histopathologic subtype (increased OR for ALM, and decreased OR for NM and other) (Table S1).

**Fig. 3:**
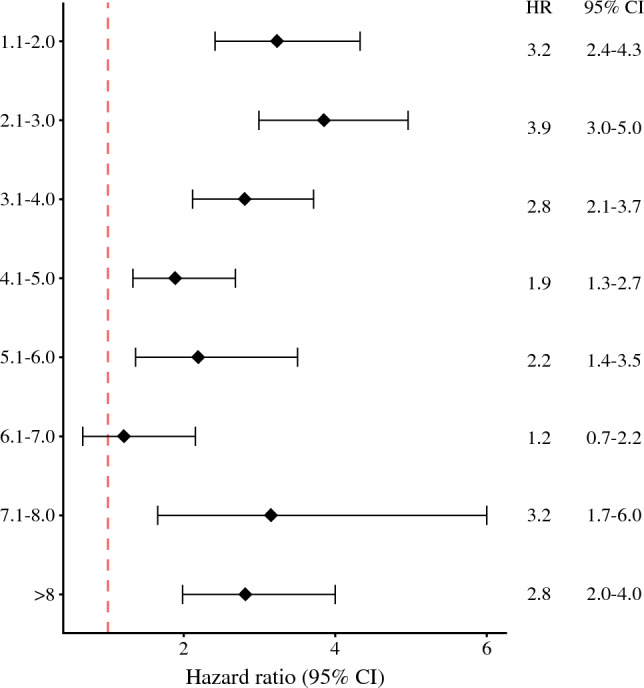
Hazard ratio for melanoma-specific survival of sentinel lymph node (SLN)-positive patients as stratified by Breslow thickness

When the data were stratified by Breslow thickness subgroups, the prognosis for the patients with a positive SLN status was consistently worse than for those with a negative SLN status (Table S2). In addition, the hazard ratio showed no significant change in magnitude with increasing Breslow thickness (Fig. 3).

When the patients were stratified by T stage, SLN status was a significant predictor for MSS. In particular, the HR for MSS among the SLN-positive patients was 2.4 (95% CI 1.6–3.5; *p* < 0.0001) for the pT4a subgroup and 2.0 (95% CI 1.6–2.5; *p* < 0.0001) for the pT4b subgroup.

When the multivariable analysis included SLN positivity and Breslow thickness as interaction variables, both Breslow groups still had significant prognostic value, with an HR of 3.7 (95% CI 3.1–4.3) for a thickness of 4 mm or less and an HR of 2.3 (95% CI 1.9–2.8) for a thickness greater than 4 mm.

### Excluded Patients

The final analysis excluded 7774 patients from the original registry cohort who had not undergone SLNB. The reasons are unknown, but most likely involved thin tumors or advanced co-morbidities. In this group, 54 % (*n* = 4.71) were male and 46% (*n* = 3603) were female. The median age was 77 years (IQR, 65–85 years), with 43% (*n* = 3325) of the patients older than 80 years. The median Breslow thickness was 2.6 mm (IQR, 1.5–5.0 mm). In the excluded group, 32% (*n* = 2467) of the patients had a Breslow thickness greater than 4 mm, and 10% (*n* = 778) of the patients had a Breslow thickness greater than 8 mm. The 5- and 10-year MSS rates for the excluded group with a Breslow thickness greater than 4 mm were respectively 81% (95% CI 80–82%) and 74% (95% CI 73–76%).

## Discussion

In this study using real-world data from the large Swedish prospective population-based registry SweMR, we verified that the outcome of SLNB continues to hold significant prognostic value also for patients with thick (> 4 mm, stage pT4) melanoma. Furthermore, the magnitude of the HR for MSS experienced by the subgroup with melanoma thicker than 4 mm is similar to that for patients with intermediate-thickness melanoma (1–4 mm, stage pT2–pT3). Our results showed a reduction in absolute MSS survival of 23% (92% vs 69%; HR 4.0; 95% CI 3.6–4.5; *p* < 0.0001) for the SLN-positive versus the SLN-negative patients in the overall cohort (pT2–pT4).

In addition to SLN status, our analysis showed that Breslow thickness, primary tumor ulceration status, and age were significant independent predictors of MSS. These findings are consistent with both the current AJCC classification system and previously published data.^11,12^

Gyorki et al.^13^ have previously presented a study of 217 patients with primary pT4 (> 4 mm) melanoma who had SLNB performed. Their cohort was predominantly male (69 %) with a median age of 64 years, a median Breslow thickness of 6 mm, and a SLN positivity rate of 36%. Multivariable analysis showed that SLN status was the most significant prognostic factor for overall survival (OS) (HR 2.88; 95% CI 1.75–4.73), followed by primary tumor ulceration status. They reported a 5-year OS of 45% for the SLN-positive patients compared with a 5-year MSS of 57% (95% CI 53–63%) for the group with a Breslow thickness greater than 4 mm and SLN positivity in our cohort. Although our chosen end points differ in terms of overall versus melanoma-specific survival, the relatively low median age of both cohorts make the figures comparable. In addition to this single-center cohort, Gyorki et al.^13^ also presented a meta-analysis of an additional 10 studies (including MSLT-1) reporting on survival outcomes for a combined total of 2104 patients with pT4 primary melanoma who had undergone SLNB.^14–22^ This meta-analysis also showed that SLN status was an independent predictor for OS in the multivariable analysis (HR 2.3; 95% CI 1.95–2.71). The findings of our current study, using data collected prospectively from a national database, are consistent with the meta-analysis.

More recently, Han et al.^23^ presented an analysis of 1235 patients with AJCC pT4 melanomas from The Sentinel Lymph Node Working Group database. The incidence of SLN metastasis was 36%. Overall, the 5-year MSS was 66%. The MSS was 75% for the SLN-negative patients and 50% for the SLN-positive patients. These authors undertook a subgroup analysis that stratified patients according to their Breslow thickness (4.0–8.0 mm [*n* = 956]; 8.1–12.0 mm [*n* = 175]; > 12.0 mm [*n* = 104]). The 5-year MSS in these three groups were 67%, 60%, and 57% respectively, with a consistently worse prognosis for the SLN-positive patients in all three groups. The single strongest prognostic marker for MSS was SLN status in all the thickness groups. The authors also identified tumor thickness, male gender, lymphovascular invasion, microsatellitosis, and the acral lentiginous and nodular melanoma histopathologic subtypes as predictive of SLN status.

The SLNB procedure can be seen as both therapeutic and prognostic, but currently, much focus lies in identifying patients that should be recommended to undergo adjuvant systemic treatment. For patients with completely resected stage III disease, pivotal phase 3 adjuvant trials have shown robust effects in terms of recurrence-free survival (RFS) for immunotherapy with a PD-1 inhibitor versus placebo, with an HR of 0.59 (95% CI 0.49–0.70) in the KEYNOTE-054 trial,^24^ as well as for targeted therapy with BRAF/MEK inhibitors, with an HR of 0.51 (95% CI 0.42–0.61), in the Combi-AD trial.^25^ However, patients with high-risk stage II (IIB/C) melanoma actually have a similar or worse survival compared with patients who have stage IIIA/B melanoma.^11^

The recently published KEYNOTE-716 trial showed a significantly longer RFS also for patients with stage IIB/C melanoma receiving adjuvant PD-1 inhibition than for those receiving a placebo (HR 0.65; 95% CI 0.46–0.92).^26^ Notably, none of the trials to date have shown any benefit in overall survival, and these data are eagerly awaited.

However, these findings also raise questions concerning the role of SLNB. Is there really a point in performing the procedure if the patient will receive adjuvant treatment anyway, especially patients with high-risk T4 tumors? We argue that SLNB still has a role, for three main reasons. First, in all the adjuvant trials, an SLNB procedure was mandated. Second, the MSLT-1 trial showed an improved disease-free survival (DFS), with a hazard ratio of 0.76 in favor of SLNB, although the gain was mainly in regional recurrences.^8^ Third, and most relevant to the data presented in this report, SLNB provided valuable information also in the group of patients with T4 tumors, with an HR for MSS ranging between approximately 1.4 and 3.5. This is important information that should be incorporated into an informed discussion with patients when a clinician is recommending adjuvant treatment.

The strength of our current study was the fact that the data were derived from a large population-based cohort, prospectively collected on a national scale, with all centers treating melanoma in Sweden contributing to the real-world dataset. This study represents the largest dataset of pT4 melanoma analyzed to date (1943 patients), based on almost all cutaneous melanoma patients treated in Sweden since 2007, with updated individual MSS. The results can therefore be considered most reliable.

We also performed an analysis of the patients with pT4 melanomas who did not undergo SLNB, and notably, these patients had a better MSS than the SLNB-negative group. The most likely explanation for this is the high level of competing risks in this population, meaning that these patients who did not undergo SLNB likely had advanced co-morbidities and therefore died of other causes before the time when they would have died of their melanoma. It is also important to note that our cohort was drawn from a generally fair-skinned population, and these results may not be representative for populations with melanin-rich skin. Furthermore, the results of our MSS analysis did not take into account the modern treatment paradigm of adjuvant systemic therapy for SLN-positive patients, which may have been offered to some patients in this cohort more recently.

In summary, the findings of this large national-registry study confirm that SLN status in pT4 melanoma patients has significant and important prognostic implications for MSS. In light of our findings, we recommend that clinical guidelines be updated to reflect the prognostic value of SLN status also for patients with thick melanomas (> 4 mm).

### Supplementary Information

Below is the link to the electronic supplementary material.

Supplementary file1 (DOCX 51 kb)
