# Supplementary material for: Prognostic Significance of Sentinel Lymph Node Status in Thick Primary Melanomas (> 4 mm)
Source: Ann Surg Oncol. 2023 Aug 14;30(13):8026–33. doi: 10.1245/s10434-023-14050-w (PMC10625939; doi:10.1245/s10434-023-14050-w)
Supplement: Supplementary file 1 — Supplementary file1 (DOCX 51 kb) [file 10434_2023_14050_MOESM1_ESM.docx]

**Supplemental data**

**Figure S1a-b.** Melanoma-specific survival of patients with **a)** negative SLNB and **b)** positive SLNB, as stratified by Breslow thickness.

**a)**

**b)**

**Table S1.** Multivariable logistic regression analysis of predictive factors for positive SLNB status in T4 patients (n=1943).

| **Variable** | **OR (95% CI)** | **n (negative SLNB)** | **n (positive SLNB)** |
| --- | --- | --- | --- |
| **Intercept** | 0.9 (0.5-1.6) | 1275 | 668 |
| **Tumor thickness (mm)** | |  |  |
| 4.1-5.0 | 1 (ref) | 522 | 243 |
| 5.1-6.0 | 0.9 (0.7-1.2) | 262 | 112 |
| 6.1-7.0 | 1.1 (0.8-1.6) | 148 | 84 |
| 7.1-8.0 | 1.5 (1.0-2.2) | 84 | 61 |
| >8.0 | 1.2 (0.9-1.6) | 259 | 168 |
| **Sex** |  |  |  |
| Men | 1 (ref) | 773 | 432 |
| Women | 0.8 (0.7-1.0) | 502 | 236 |
| **Age (years)** |  |  |  |
| <40 | 1 (ref) | 44 | 41 |
| 40-59 | 0.7 (0.4-1.1) | 215 | 163 |
| 60-69 | 0.3 (0.2-0.6) | 346 | 145 |
| 70-79 | 0.4 (0.2-0.7) | 469 | 226 |
| ≥80 | 0.4 (0.2-0.6) | 201 | 93 |
| **Tumor site** |  |  |  |
| Extremities | 1 (ref) | 548 | 275 |
| Head/neck | 0.5 (0.4-0.8) | 150 | 42 |
| Trunk | 1.1 (0.9-1.4) | 542 | 309 |
| Palm/subungual | 1.4 (0.7-2.5) | 33 | 39 |
| **Tumor ulceration** |  |  |  |
| Absent | 1 (ref) | 479 | 174 |
| Present | 1.8 (1.4-2.2) | 768 | 479 |
| **Clark's level of invasion** | | 1.6 (1.1-2.4) |  |
| II-III | 1 (ref) | 131 | 57 |
| IV | 1.1 (0.8-1.6) | 767 | 379 |
| V | 1.6 (1.1-2.4) | 350 | 214 |
| **Histopathologic subtype**^a^ | | 0.9 (0.5-1.6) |  |
| SSM | 1 (ref) | 265 | 186 |
| ALM | 1.9 (1.0-3.9) | 21 | 38 |
| NM | 0.7 (0.5-0.8) | 762 | 348 |
| Other | 0.5 (0.4-0.8) | 190 | 73 |

(^a^SSM = superficial spreading melanoma, ALM = acral lentiginous melanoma, NM = nodular melanoma)

**Table S2.** Univariable Cox regression analysis of melanoma-specific survival by SLNB status as stratified by Breslow thickness and T stage.

| **SLNB status** | **Negative** | **Positive** | **n (negative SLNB)** | **n (positive SLNB)** |
| --- | --- | --- | --- | --- |
|  |  | (HR, 95% CI) |  |  |
| **Breslow thickness** |  |  |  |  |
| 1.1-2.0 | 1 (ref) | 3.2 (2.4-4.3) | 4846 | 561 |
| 2.1-3.0 | 1 (ref) | 3.9 (3.0-5.0) | 1602 | 470 |
| 3.1-4.0 | 1 (ref) | 2.8 (2.1-3.7) | 764 | 305 |
| 4.1-5.0 | 1 (ref) | 1.9 (1.3-2.7) | 522 | 243 |
| 5.1-6.0 | 1 (ref) | 2.2 (1.4-3.5) | 262 | 112 |
| 6.1-7.0 | 1 (ref) | 1.2 (0.7-2.2) | 148 | 84 |
| 7.1-8.0 | 1 (ref) | 3.2 (1.7-6.0) | 84 | 61 |
| > 8 | 1 (ref) | 2.8 (2.0-4.0) | 259 | 168 |
| **T stage** |  |  |  |  |
| T2a | 1 (ref) | 3.3 (2.3-4.8) | 3883 | 411 |
| T2b | 1 (ref) | 2.8 (1.7-4.6) | 807 | 137 |
| T3a | 1 (ref) | 4.7 (3.4-6.2) | 1312 | 372 |
| T3b | 1 (ref) | 2.7 (2.1-3.4) | 976 | 385 |
| T4a | 1 (ref) | 2.4 (1.6-3.5) | 479 | 174 |
| T4b | 1 (ref) | 2.0 (1.6-2.5) | 768 | 479 |
